# Thermomechanical Properties of High Oleic Palm Oil Assessed Using Differential Scanning Calorimetry, Texture Analysis, Microscopy, and Shear Rheology

**DOI:** 10.3390/gels9100798

**Published:** 2023-10-04

**Authors:** Victor Cedeno-Sanchez, Melissa Perez-Santana, Devanshu Mehta, Scarlett Godinez, Liwei Gu, Victoria M. Miller, Andrew J. MacIntosh

**Affiliations:** 1 Food Science and Human Nutrition Department, University of Florida, Gainesville, FL 32611, USA; 2 Department of Chemistry, University of Florida, Gainesville, FL 32611, USA; 3 Department of Materials Science and Engineering, University of Florida, Gainesville, FL 32611, USA

**Keywords:** high oleic palm oil, microscopy, rheology, fatty acid profile, texture, DSC

## Abstract

Standard Palm Oil (SPO) is widely used as a food ingredient partially due to its unique thermophysical properties. However, the American Heart Association recommends a saturated fat consumption of <5% of the caloric intake per day. The *OxG* Palm hybrid yields oil known as “palm oil with a higher content of oleic acid” (HOPO), with <35% SFA and >50% oleic acid. Characterizing novel high oleic oils is the starting point to find processes that can functionalize them such as oleogelation. This study compared the thermophysical properties of HOPO to SPO using Differential Scanning Calorimetry, shear rheology, polarized light microscopy, and texture analysis to characterize the differences between these oils. HOPO had a lower onset crystallization temperature (Δ7 °C) and its rheological behavior followed similar trends to SPO; however, large viscosity offsets were observed and were correlated to differences in crystallization temperatures. The maximum peak force of SPO was an order of magnitude higher than that of HOPO. Overall similar trends between the oils were observed, but differences in firmness, crystal morphology, and viscosity were not linearly correlated with the offset in crystallization temperature. This study quantified differences between these oils that will better enable industry to use HOPO in specific applications.

## 1. Introduction

Edible fats and oils play a key role in the human diet, providing energy, essential fatty acids for cellular development, and delivery of liposoluble phytonutrients [1]. As an ingredient, fats can improve the sensory attributes of foods, such as the texture and mouthfeel. Currently, the most consumed fat in the world is Standard Palm Oil (SPO), from the species *Elaeis guineensis*, largely due to its versatility as a food ingredient [2] and highest yield per hectare when compared to any other major oil-bearing crop [3]. The typical saturated fatty acid (FA) content in SPO (~50%) is responsible for many of its physical properties including its solid structure at room temperature and firmness [4]. The fatty acid profile of SPO makes it appropriate for inclusion in oleogels, shortenings, and margarine, as these require profiles that are solid at room temperature [2,4]. However, a consumption of <5% saturated fatty acids (SA) in the daily diet of an average adult is recommended by the American Heart Association (AHA) [5]. The AHA further recommends the consumption of alternative oils that are higher in mono- and polyunsaturated fatty acids, which have been shown to reduce the incidence of cardiovascular disease [5,6,7]. Palm oil varieties with higher contents of oleic acid (a monounsaturated acid) have recently been developed [8]. However, there is currently a scarcity of published information concerning their properties and applications. This research characterized and compared oil from a hybrid palm, higher in monounsaturated fatty acids than standard palm oil (SPO), to determine the differences in thermophysical and structural properties.

In 2019, the Codex Alimentarius included a new definition for a “palm oil with a higher content of oleic acid” in the standard for named vegetable oils. Grown primarily in Colombia, Ecuador, and Brazil, this oil is obtained from the interspecific hybrid *Elaeis oleifera X Elaeis guineensis (OxG)* palm [9]. This palm oil is higher in oleic acid (HOPO) and is potentially beneficial to consumers due to the higher concentration of monounsaturated oleic acid (53.5–55.25% of total FA) than SPO (37% of total SA), resulting in a lower concentration of saturated fatty acids for HOPO (typically 30%). It should be noted that the concentration of fatty acids (a critical parameter in fat crystallization) varies in commercial oils due to the effects of seasonal variability and agricultural practices [10]. A higher content of saturated fatty acids is associated with a higher melting point of the fat [11]. Furthermore, most of the oleic acid in HOPO (64.7–66%) is in the sn-2 position of the triglyceride, where it is most likely to be absorbed by the body [12]. HOPO also has higher reported concentrations of tocotrienols [13] and carotenoids [14] when compared to SPO, which are nutrients of interest for proper bodily functions. The position of the fatty acid chain in the triglycerides not only affects the way it is absorbed by the body, but also the physical properties of the fat. HOPO has less completely saturated triglycerides (1.6%) than SPO (8.5%), which may affect the room temperature stability of the oils [12]. As the solid fat content (SFC) is known to impact the functional applications such as the spreadability of the fat [15], it is necessary to assess the impact of the fatty acid profiles of HOPO and SPO on their thermal and rheological properties.

The crystallization process of fats and oils is dependent on both intrinsic and extrinsic factors. Intrinsic factors include the fatty acid profile, triglyceride composition, and the presence of naturally occurring minor components. Minor components include free fatty acids (FFA), monoglycerides, diglycerides, and impurities. Major extrinsic factors include the cooling rate, degree of undercooling, annealing time, and shear rate during the formation of the crystalline structure. Differential Scanning Calorimetry (DSC) is commonly used to study exothermic and endothermic reactions caused by temperature changes under constant cooling and heating regimes. Phase transitions are identified by deviations from the baseline heat requirement to change temperature. Once crystallized, the firmness of the oil is an additional parameter that varies with temperature and solid fat content [16], which describes resistance to force. Penetration tests are common techniques to assess the firmness of commercial fats and margarine due to their ease of use and repeatability [17].

Numerous researchers have correlated the physical properties of fats to their crystalline structure [16,18]. Microscopy is used to characterize fat crystals in terms of size, shape, and abundance, each affecting the mechanical properties and mouthfeel of the final products [19]. The size and morphology of the crystal shapes depend upon the fatty profile, cooling rate, and shear rate, among other parameters [20,21]. Other techniques include pulsed nuclear magnetic resonance (pNMR), a technique to quantify the solid fat content, and X-ray diffraction, which describes the type of polymorph of the fat crystals for the same purpose [22]. For industrial applications, oils are routinely exposed to different cooling and heating regimes combined with ranging shear rates, which results in changes to crystal structure, size, orientation, aggregation, and rearrangement [23].

Currently, there is limited knowledge on the crystallization kinetics and physical properties of HOPO and its products [24]. Publications have addressed the use of HOPO in oleogel formulations using mono and diglycerides [25] and some of these oleogels perform identically to SPO oleogels in the baking of cookies [26]. However, a general characterization of HOPO is needed to understand this oil as an ingredient and enable the expansion of its use to multiple applications. The focus of this study was to use several common methods to compare the properties of SPO to HOPO. The effect of intrinsic and extrinsic factors was studied by comparing thermal, physical, optical, and rheological properties.

## 2. Results and Discussion

### 2.1. Fatty Acid Methyl Esters (FAME) by GC-FID

The fatty acid profile obtained in this study for HOPO was 27.8% palmitic acid (C16:0), 2.8% stearic acid (C18:0), 55.5% oleic acid (C18:1), and 11.9% linoleic acid (C18:1). These results matched previously published data for HOPO shown in Table 1 for palmitic (C16:0), stearic (C18:0), oleic (C18:1), and linoleic (C18:2) acids, representing more than 95% of the fatty acids. Those under 1% *w*/*w* were not reported. The literature values for SPO are shown in Table 1 for comparison.

It was confirmed that HOPO had a lower concentration of palmitic and stearic fatty acids when compared to SPO (the most representative saturated fatty acids found in palm oil); this was expected, and it was also observed by Mozzon et al. [12]. Consequently, HOPO had a considerably higher content of oleic acid, which is the monosaturated fatty acid of nutritional interest. It also contained a slightly higher concentration of linoleic acid when compared to SPO.

### 2.2. Differential Scanning Calorimetry

**Figure 1:**
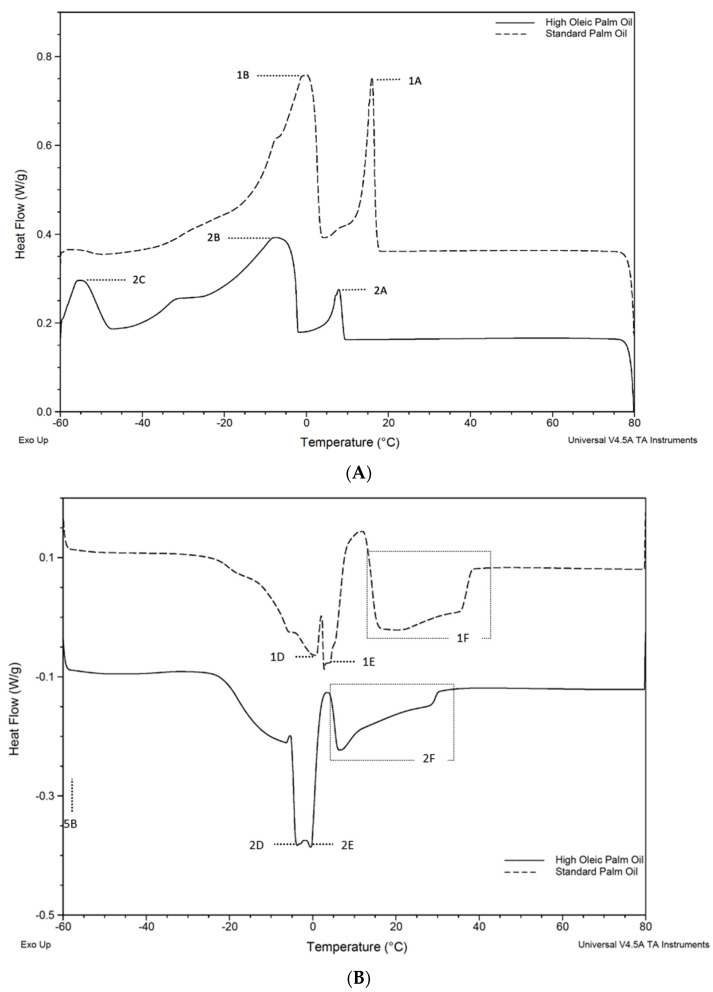
DSC thermograms of High Oleic Palm Oil (HOPO) and Standard Palm Oil (SPO) at 10 °C/min. For clarity, the SPO energy flow is offset by +0.2 W/g sample. (**A**) Labeled peaks (A–C) correspond to major component crystallization events. (**B**) Peaks D–F correspond to melting events.

The results from this analysis showed SPO’s thermogram consists of two-phase transition zones for the crystallization and melting stages. This was consistent with the thermograms reported in previous studies [29]. The crystallization and melting curves obtained by the DSC are shown in Figure 1A,B, respectively. The first cycle started at T = 80 °C and was cooled to −60 °C at a constant rate; this was observed following a counterclockwise reading. Overall, it seems like similar crystallization and melting events occur in both oils with a ΔT of around 7 °C (Table 2 and Table 3).

While most of the work in this study focused on temperatures above 0 °C (in the common range of food industry applications), the first large melting peak for HOPO had an onset of −5.45 °C which ends at 3.5 °C, while for SPO, the peak extends up to 10 °C. This indicates that the lower-melting fraction of the HOPO will be almost completely melted at temperatures below 0 °C. The melting profile from the thermogram showed complete melting at 30.03 °C for HOPO and 37.99 °C for SPO (see Table 3). The full transition from a crystalline structure to fully melted in SPO has been reported to occur at 36.32 °C [30].

In the cooling diagram, the reduced energy required to transition at E and F likely corresponds to the lower concentration of di-saturated and fully saturated TAGs (~65% vs. ~53%) in HOPO when compared to SPO. These results were consistent with the findings of Mozzon et al. [12], who also showed that ‘hybrid palm oil’ had an oleic acid content higher than crude palm oil. The thermal transitions are aligned with results for HOPO in [8,25]. Based on the onset temperature and peak crystallization points observed in both oils, the mechanical, rheological, and visual characteristics were tested at 20 °C, 15 °C, 10 °C, and 5 °C.

### 2.3. Texture Analysis

**Figure 2:**
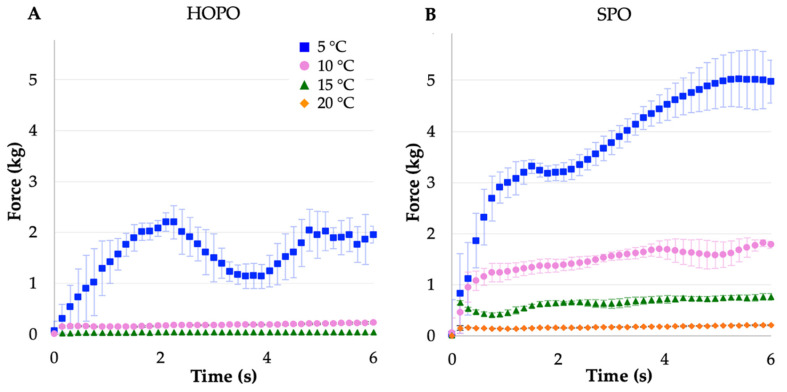
Penetration force over time for (**A**) HOPO. (**B**) SPO hardness at 5, 10, 15, and 20 °C. Error bars indicate standard deviation. *n* = 4.

As shown in Figure 2, at lower assessment temperatures, the force required to puncture increased, likely due to the amount of solid (crystallized) fat. At 5 °C, SPO exhibited plastic deformation, characterized by the roughly linear shape of the response to the axial force applied by the probe, with a positive peak force of 5.06 kg (Table 4). At this temperature, the fat behaved as a semisolid, deforming without showing an ultimate yield point. The most noticeable increase in both peak force and work of penetration was between 10 and 5 °C for both oils. At 5 °C, HOPO showed a positive increment in force followed by a decrease, this may suggest the crystallization process started from the edges of the vessel due to the direction of the heat transfer from the surface next to the water bath to the center, creating a crust and a soft core. A longer equilibration time may result in an only positive slope curve; the highest positive peak force was 2.5 kg (Figure 2A). It was then noted that at 5 °C, the first crystallization peak of HOPO had not ended, while for SPO it was already finished; therefore, there was a portion of low-melting fatty acids in HOPO that may not have crystallized to create a solid network that increases the hardness of the oil, which could have been observed if an equilibrium was present at around −3 °C.

As shown in Table 4, at 15 and 20 °C, the SPO stiffness plateaued at values around 0.8 and 0.2 kg, respectively (see also Figure 2B). At 10 °C, HOPO exhibited a very small resistance to the probe compared to SPO, leading to a plateau around 0.20 kg. At 15 °C, HOPO stiffness values were barely higher than the threshold detected by the instrument between 0.018 and 0.036 kg. When compared to SPO, the overall stiffness values were lower in HOPO, possibly due to a lower SFC at the same temperature and probable small and dispersed crystalline structures, as discussed in the following section. It was not possible to measure HOPO’s stiffness at 20 °C due to its liquid behavior at this temperature.

Even when similar phase transitions occur at 7 °C apart in the thermograms (Figure 1), the stiffness of HOPO is not similar to that of SPO at 7 °C difference; therefore, it is hypothesized that other factors at the given temperatures had major roles in the stiffness of the oil, such as triglyceride composition and the effect on the cooling rate with crystalline structure formation from nuclei composed of saturated fatty acids.

### 2.4. Polarized Light Microscopy

**Figure 3:**
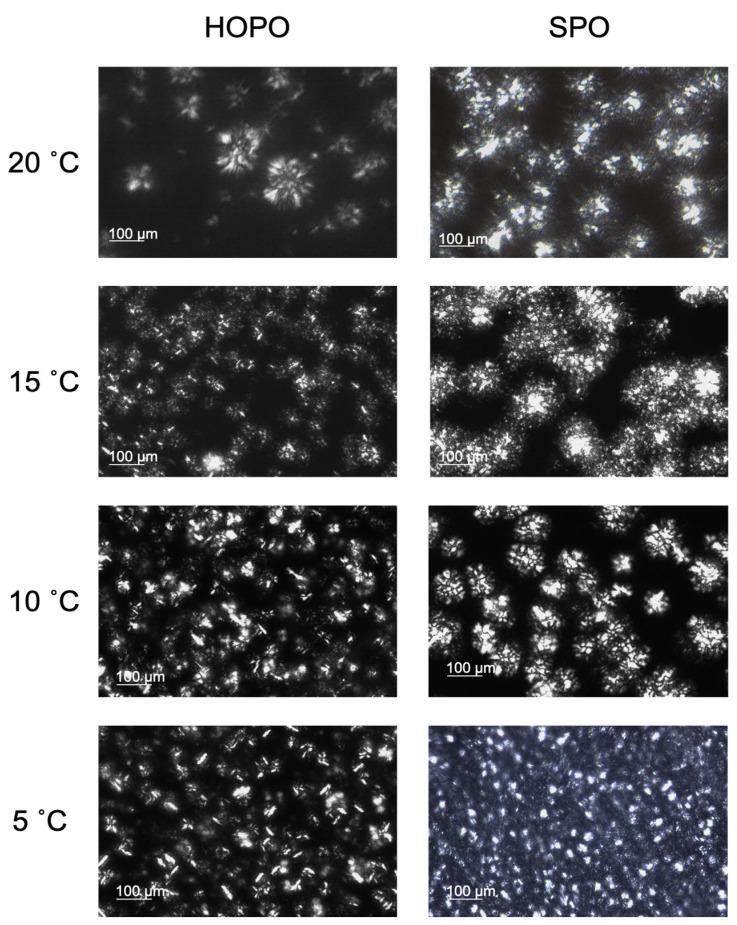
Images of the microstructure of HOPO and SPO at 20 °C, 15 °C, 10 °C, and 5 °C. The bar represents 100 µm.

The characterization of the crystalline microstructure of fats can help better assess the mechanical properties of fats. In polarized microscopy, fat crystals appear as white and the liquid oil in the background as black. The samples were crystallized at 5, 10, 15, and 20 °C for 24 h and images were taken in duplicate (Figure 3).

Spherulites were the most common structures identified in both oils. For HOPO, structures ranged from 18.1 ± 1.1 µm at 20 °C to 8.6 ± 1.3 µm at 5 °C and between 12.9 ± 1.1 µm at 20 °C and 8.8 ± 1.2 at 5 °C for SPO. The ideal spreadable fat consists of small (<30 µm) and largely dispersed crystals [31]. The β polymorph is mostly associated with spherulites sized between 20 and 30 µm [19]. At 15, 10, and 5 °C, irregular shapes were prevalent, dominating the microstructure, making it difficult to accurately measure the size of the crystals or describe the morphology. In the case of SPO, the microstructure is dominated by regular spherulite crystals at all temperatures, with the packing increasing as the temperature dropped. The crystalline transition forms can be modified through shear and cooling rates, resulting in changes to the physical properties of the oil. This is key for operating conditions to produce semi-solid industrial fats [32]. Changes include graininess, stiffness, or phase separation.

### 2.5. Shear Rheology

Shear rheology has long been utilized to describe the first and second crystallization temperatures of palm oil under shear by notable jumps in viscosity at constant cooling rates [23]. The same study noticed lower viscosity plateaus at higher shear rates at constant cooling rates, mainly due to the type of crystalline aggregates formed. Similar results were obtained by Moelants et al. [33]. This study used a constant shear, since stable nucleation sites were found to be promoted under constant shear [34].

**Figure 4:**
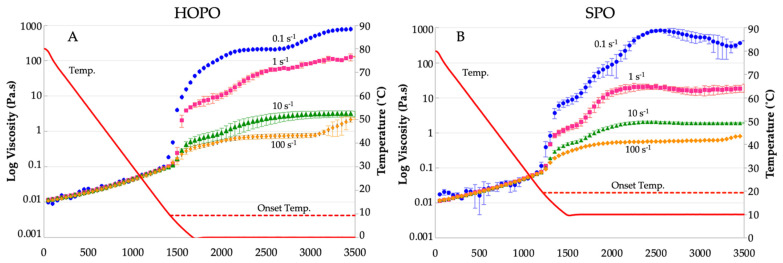
Viscosity at shear rates of 0.1, 1, 10, and 100 s^−1^ for (**A**) HOPO and (**B**) SPO. *n* = 2 trials were completed, and the error bars indicate standard deviation. The horizontal dotted line represents the onset crystallization temperature of the oil as observed via DSC. The X axis represents time in seconds.

The dynamic viscosities vs. temperature and shear rates (0.1, 1, 10, and 100 s^−1^) are shown in Figure 4A for HOPO, and in Figure 4B for SPO, at a constant cooling rate of 3 °C/min. Both oils exhibited viscosity reductions upon increases in shear. The similarity in the trends in viscosity changes in HOPO vs. SPO with shear and temperature suggests that HOPO can be targeted to behave similarly to SPO, with crystallization processes expected to take place with a delta of −10 °C and with minor adjustments to the shear rate. The induction time (required time for the oil to start crystallization) for SPO was shorter and crystallization started to occur near 19.3 °C, closer to the onset temperature of 17.41 °C identified in the DSC thermograms. For HOPO, this temperature was around 9.3 °C, almost the same onset temperature identified in the DSC thermograms. All shear rates resulted in distinct viscosity curves, with a progressive lessening of the effect towards higher shear rates. A low shear led to early promotion of crystallization, observed for HOPO and SPO. HOPO registered an initial crystallization at 1250 s at 0.1 s^−1^ and 1350 s for higher shear rates; SPO crystallization started at 1150 s at 0.1 s^−1^ and 1200 s for higher shear rates. The viscosity of HOPO was higher than SPO at lower shear rates (0.1 and 1 s^−1^) at the offset of −10 °C. In [32], similar behavior was described for cocoa butter, for which a low shear reduced the onset time and a high shear delayed it, supporting the phenomenon of generation of viscous heat. Considering that these lower shear levels resulted in higher viscosity values for both oils, it was likely that aggregation was promoted; however, observing the SPO thermogram, there is a shoulder at the end of the first crystallization peak, and this shoulder develops at around 7 °C. Since the rheology was maintained at 10 °C, is it likely that the higher-melting fat portion that acts as nuclei for crystal formation was partially melted and was not able to grow. This is in contrast to HOPO, which did not exhibit any shoulder in the melting thermogram at 0 °C; all higher melting template crystals had formed and could aggregate better. In [23], this behavior was explained for palm oil due to large crystal structures being able to develop at higher shear rates, creating fat aggregate suspensions. The viscosity of HOPO sheared at 1 s^−1^ plateaus at two orders of magnitude higher than that of shears of 10 s^−1^ and 100 s^−1^.

HOPO is a fat that can be used in multiple applications if the characteristics of SPO are needed with a lower saturated fat level. A converging point of unsaturated fat which is in a semi-solid state can be achieved by methods such as interesterification or oleo gelation, the latter being a low-cost approach enabling the use of health-beneficial oils in applications that require a matrix with no flow in the steady-state, i.e., a gel. Several studies have tried to correlate oil characteristics with the rheological and mechanical behavior of oleogels. As reviewed by Li et al. [35], viscosity may have opposing effects on oleogel hardness; in the case of olive, corn, sunflower, and flaxseed oil gelled by β-sitosterol + stearic acid, a higher oil viscosity resulted in a lower oleogel hardness. However, in the case of castor, corn, cod, EVOO, flaxseed, peanut, and sunflower oil gelled by monoglycerides, the viscosity of the oil was positively correlated with the firmness and rheological parameters of the oleogel. Even with the knowledge of the mechanical and rheological behavior of the oil to be used in an oleogel, for which HOPO is a candidate, the effect of this oil will depend on the type of gelator or structuring agent and their relation under different processing conditions. For the purpose of formulating oleogels with HOPO, general rules can be followed, as stated by Co and Marangoni [36]. These rules include that the gelator should be relatively insoluble in HOPO (the solvent) and that there should be low HOPO–gelator interactions to allow gelator–gelator self-assembly and crystallization. However, the gelator cannot be too soluble otherwise it will just dissolve in HOPO.

## 3. Conclusions

A comparison between the rheological and thermal characteristics of standard palm oil and the high oleic variety was established and related to their fatty acid composition. The FAME analysis showed significant differences in monounsaturated FA, mainly in the oleic acid content, which is higher in HOPO (53.92% vs. 40.15%), and saturated fatty acids, represented as palmitic acid (29.92% vs. 43.52%).

The effect of the FA profile on the melting/cooling behavior during DSC analysis was observed for both oils. The thermograms had similar shapes; however, a ~7 °C offset in the crystallization and melting profiles was identified. The onset crystallization temperature started at 7.90 °C in HOPO, whereas it occurred at 16.45 °C in SPO. HOPO completely melted at 29 °C and SPO at 37 °C. These findings are important to establish the optimal crystallization temperatures for HOPO and how these will affect its mechanical and rheological characteristics. Major crystallization events occurred at 16.45 °C, 14.05 °C, 4.64 °C, and −1.18 °C in SPO and were consistent with the literature. The corresponding values for HOPO were 7.9 °C, 5.81 °C, −1.27 °C, and −8.11 °C, which offset the maximum usable temperature of HOPO.

A texture analysis for the oils near major crystallization temperatures found the highest stiffness value to be 2.495 ± 0.182 kg at 5 °C for HOPO and 5.064 ± 0.546 at 5 °C for SPO, with the difference that SPO deformed as force was applied, while HOPO was more brittle and broke at the maximum stiffness.

The microstructure of HOPO was temperature-sensitive with well-defined spherulites at 20 °C, but irregular and ambiguous structures at 15 °C and 10 °C. The microstructure of SPO did not experience major structural changes with the decrease in temperature. Under different cooling regimes, the viscosity profiles were similar between the oils at higher shears; however, there was a shift in the induction time of nearly 4 min for crystallization, occurring at around 6–8 °C in HOPO at high shears, 10 °C less than for SPO.

This study expands the understanding of the physical properties of HOPO, suggesting that low-temperature applications can harness the soft but viscous texture of HOPO while taking advantage of it being a nutritious alternative to oils with higher saturated fatty acid concentrations. Additionally, it was shown how the processing conditions, shear, and cooling temperatures play a key role in the applications of HOPO as an ingredient. Given its low viscosity at room and refrigerator temperatures, HOPO can be used as a fluid fat component in the making of functional fats such as shortenings, oleogels, soft margarines, or spreads, which require a soft texture at refrigerated storage conditions.

## 4. Materials and Methods

### 4.1. Materials

Refined, bleached, and deodorized (RBD) high oleic palm oil (HOPO) from the interspecific hybrid *E. Guineensis* and *E. Oleifera OxG* was supplied by Thin Oil Products (Weston, FL, USA). The specifications of HOPO are summarized as an Iodine Value (IV) of >67, a melting point of 27 °C, and moisture of <0.05%. Refined, bleached, and deodorized standard palm oil (SPO) from *E. Guineensis* was supplied by Catania Oils (MA, USA). The specifications of SPO are summarized as an Iodine Value (IV) of >52.7 and a melting point of 39.7 °C.

### 4.2. Fatty Acid Methyl Esters (FAME) by GC-FID

Some variability in the fatty acid profile for palm oil in the literature is expected as regional variability has been identified [12,37]. Due to historical variability in the fatty acid profile of HOPO, the fatty acid composition of the oils used was determined by Gas Chromatography-Flame Ionized Detector of Fatty Acid Methyl Esters according to the AOCS method Ce1b-89 [38].

### 4.3. Differential Scanning Calorimetry

To examine the crystallization and melting events under static (non-sheared) conditions, a Differential Scanning Calorimeter (DSC) Q1000 (TA Instruments) was used. Triplicate samples of each oil weighing 8–9.8 mg were weighed into aluminum pans to the nearest 0.1 mg and tightly sealed. An empty, hermetically sealed aluminum pan was used as a baseline. The experiment was conducted in a controlled helium atmosphere chamber. Samples were equilibrated at 80 °C for 10 min, crystallized at 10 °C/min at −60 °C (held for 10 min), and melted at 5 °C/min at 80 °C (held for 10 min) as described by the AOCS Official Method Cj 1-94 [39]. TA Universal Analysis software was used for analysis of endothermic and exothermic peaks.

### 4.4. Texture Analysis

A standard puncture test from Texture Technologies Corp [40] was conducted on the solid oils at various temperatures to assess the stiffness of each solid fat. Samples were melted at 80 °C in a water bath, placed in glass vials (n = 4), and then transferred to a set of water baths at 45 °C, 40 °C, 35 °C, and 30 °C, respectively. Upon equilibrium, samples were transferred to a second set of water baths at 20 °C, 15 °C, 10 °C, and 5 °C, respectively, and conditioned for 24 h. The second water bath was used to achieve the same temperature difference (T_final bath1_ − T_final bath2_ = 25 °C) and therefore simulate similar cooling rates for each of the final temperatures. The 24 h conditioning facilitated the formation and stabilization of the crystals. The tests were performed immediately after removing the samples from each water bath. The puncture test was performed on a TA-XTplus texture analyzer (Stable Micro Systems, Scarsdale, NY, USA) equipped with a 6 mm diameter cylindrical probe (probe at room temperature). The probe was inserted into the sample at 2 mm/s to a depth of 10 mm, and removed at 2 mm/s. The maximum force encountered during the insertion was recorded as the peak force, while the area under the force vs. time curve was recorded as the work of penetration. As per the method, the force area is correlated to the firmness of the sample. The test was replicated in *n* = 4 vials under identical conditions.

### 4.5. Polarized Light Microscopy (PLM)

To examine the morphology and size of fat crystals at various crystallization temperatures, the samples were observed using a Nikon Eclipse Ci (NY, USA) microscope equipped with polarized filters, similar to the methods described by Tang and Marangoni [41]. To prepare the samples, HOPO and SPO were heated to 70 °C, then 10 µL of the melted oils was placed on pre-heated slides at 65 °C and covered with a coverslip at the same temperature. Samples were left to rest on a temperature-controlled plate at 20 °C, 15 °C, 10 °C, and 5 °C in a dry air (humidity < 5%) chamber to prevent condensation. This provided equal cooling rates for each sample, which was then held isothermally for 24 h prior to observation. Samples were prepared at each corresponding temperature and assessed immediately after removal from the isothermal chamber. Each slide was observed at 100×. Nikon Dsi software (NY, USA) was utilized to capture the images and measure the cross section of all sphere-like structures by using the 100 µm scale as a reference. In this analysis, all crystals were assessed for each image shown in Figure 3. Therefore, some conditions had a considerably larger number of crystals assessed than others.

### 4.6. Shear Rheology

Shear rheology was used to assess crystallization under sheared conditions, similar to the methods described in [23]. The dynamic viscosity of each oil was measured using a Discovery series HR-1 rheometer (DE, USA) while the sample was cooled from a temperature of 70 °C. The liquid oil at 70 °C was contained in a concentric cylinder (cup and bob setup) to reduce measuring errors at low initial viscosities and maintain a constant thickness. The samples were maintained at 70 °C for 10 min to erase the crystal memory as described in [42], while being well below the temperatures reported to induce thermal degradation in [43]. The samples were subsequently cooled to 10 °C for SPO and 0 °C for HOPO (these being the offset temperatures of the initial crystallization peaks 1A and 2A, as measured with DSC) at a rate of 3 °C/min. The viscosity was assessed at shear rates of 0.1, 1, 10, and 100 s^−1^. Different final crystallization temperatures were chosen due to the sensitivity of the instrument (when a preliminary experiment was run for SPO at 0 °C, there was an increase in viscosity that exceeded the rheometer’s capacity, precluding reliable data acquisition); therefore, the offset temperature of the first crystallization for each oil was chosen.

### 4.7. Statistical Analysis

One-way ANOVA was used to identify differences between means with temperature as the independent variable in the texture analysis, and Tukey’s HSD test was used to determine the statistical significance. DSC onset, peak, and offset temperatures were compared using an unpaired *t*-test. The level of significance for all tests was α = 0.05. Statistical analyses were performed with GraphPad Prism 8.0 (GraphPad Software, Inc., San Diego, CA, USA).

## Figures and Tables

**Table 1:** Total fatty acid composition (% *w*/*w*) of HOPO and SPO.

Oil	% Fatty Acid (*w*/*w*)				Reference
	C16:0	C18:0	C18:1	C18:2	
SPO	41.9–44	4.1–5.2	39.4–41.3	8.9–10.5	[11,27,28]
HOPO	28.6–31.3	2.4–3.1	51.9–55.1	10.2–11.9	[11,27]

Note: the range of values for each fatty acid chain was compiled using data from the cited references.

**Table 2:** Crystallization temperatures identified by DSC.

Oil Type	Temperatures (°C)						
	A		B			C	
	T_onset_	T_peak_	T_onset_	T_peak_	T_shoulder_	T_onset_	T_peak_
SPO	17.4 ± 0.5 ^a^	16.0 ± 0.1 ^a^	3.4 ± 0.1 ^a^	0.11 ± 0.2 ^a^	−7.78 ± 0 ^a^	-	-
HOPO	9.3 ± 0.2 ^b^	7.9 ± 0.1 ^b^	−2.1 ± 0 ^b^	−7.53 ± 0 ^b^	−32.22 ± 0.1 ^b^	−48.6 ± 0.1	−55.0 ± 0.1

**Note**: Corresponding peaks are shown in Figure 1. SPO peaks are labeled 1A to 1F and HOPO peaks are labeled 2A to 2F. Different letters represent significant differences within the between rows at the *p* < 0.05 probability level (*t*-test). *n* = 3.

**Table 3:** Melting temperatures identified by DSC.

Oil Type	Temperatures (°C)					
	D	E		F		
	T_peak_	T_peak_	T_offset_	T_peak_	T_shoulder_	T_offset_
SPO	0.8 ± 0.0 ^a^	2.8 ± 0.0 ^a^	12.3 ± 0.2 ^a^	15.6 ± 0.0 ^a^	35.9 ± 0.1 ^a^	37.9 ± 0.1 ^a^
HOPO	−3.7 ± 0.1 ^b^	−0.5 ± 0.0 ^b^	3.5 ± 0.1 ^b^	6.4 ± 0.0 ^b^	28.7 ± 0.1 ^b^	30.0 ± 0.1 ^b^

**Note**: Corresponding peaks are shown in Figure 1. SPO peaks are labeled 1A to 1F and HOPO peaks are labeled 2A to 2F. Different letters represent significant differences within the between rows at the *p* < 0.05 probability level (*t*-test). *n* = 3.

**Table 4:** Stiffness (peak force) and work of penetration (W.P.) values for HOPO and SPO at different temperatures.

Temperature (°C)	HOPO		SPO	
	Peak Force (kg)	W.P (kg.s)	Peak Force (kg)	W.P (kg.s)
**5**	2.49 ± 0.2 ^a^	6054 ± 594.8 ^a^	5.06 ± 0.6 ^a^	15,642 ± 1195 ^a^
**10**	0.22 ± 0.0 ^b^	688.5 ± 39.9 ^b^	1.89 ± 0.1 ^b^	5738 ± 403.3 ^b^
**15**	0.04 ± 0.0 ^c^	112.0 ± 12.9 ^b^	0.80 ± 0.0 ^c^	2496 ± 133.5 ^c^
**20**	-	-	0.21 ± 0.0 ^d^	666.2 ± 17.9 ^d^

Different letters represent significant differences within the same column at the *p* < 0.05 probability level (Tukey’s test). *n* = 4.

## Data Availability

Data presented within the article.
